# 

**Published:** 1858-04

**Authors:** 


PUBLISHED MONTHLY.

VOL. I.] [No. 4.

### THE CHICAGO MEDICAL JOURNAL

EDITED BY

N. S. DAVIS, M. D.,

AND

W. H. BYFORD, M. D.

APRIL, 1858.

JAS. BARNET, BOOK AND JOB PRINTER, "CHRONOTYPE" OFFICE,

189 Lake Street, (Corner of Wells), Chicago, Ill.

AT $2 PER ANNUM, IN ADVANCE.

